# Strangulated Spiegel hernia: About a case and literature review

**DOI:** 10.1016/j.amsu.2021.102453

**Published:** 2021-05-28

**Authors:** Mounir Bouali, layla el attar, Khalid Elhattabi, Abdelilah Elbakouri, Fatimazahra Bensardi, Abdelaziz Fadil

**Affiliations:** a Visceral Surgical Emergency Department, Universitary Hospital Center Ibn Rochd, Casablanca, Morocco; b Faculty of Medicine and Pharmacy,Hassan II University, Casablanca, Morocco

**Keywords:** Spiegel's hernia, Case report, Surgery

## Abstract

The anterolateral abdominal Hernias are a frequent reason for consultation; Spiegel's hernia is a rare spontaneous abdominal anterolateral hernia (0.12% of abdominal hernias) for patients between 40 and 70 years old, There are risk factors such as intra-abdominal hyperpressure secondary to morbid obesity, multiple pregnancies and chronic cough. The surgery is the standard treatment; whether by raphy or prosthetic mesch. We report the case of a 42 year old male admitted to the emergency room for an occlusion syndrome due to the strangulated spiegel hernia with caecal and appendicular contents.

## Highlights

∙ Hernias of the anterolateral abdominal wall are a frequent reason for consultation;
∙Spiegel's hernia is a rare form of spontaneous abdominal anterolateral hernia (0.12% of abdominal hernias) in subjects between 40 and 70 years of age
∙There are predisposing factors such as intra-abdominal hyperpressure secondary to morbid obesity, multiple pregnancies and chronic cough, the treatment is surgical; by raphy or by prosthetic plasty.

## 1 Introduction

Anterolateral abdominal wall hernias are a frequent reason for consultation; Spiegel's hernia is a rare form of spontaneous abdominal anterolateral hernia (0.12% of abdominal hernias) in people between 40 and 70 years old [1]; it is often externalized at the weak point located at the intersection between the semilunar line and the lateral end of the arched line, In a transition zone of the posterior rectus abdominis aponeurosis [2]. We report the case of a 42 year old male admitted to the emergency room for an intestinal occlusion syndrome for strangulated spiegel hernia with caecal and appendicular contents.The aim of our work is to highlight the diagnosis difficulties and the different therapeutic modalities of this pathology. This manuscript has beenreported in line with SCARE's 2020 Criteria [3].

### 1.1 Case presentation

**Fig. 1:**
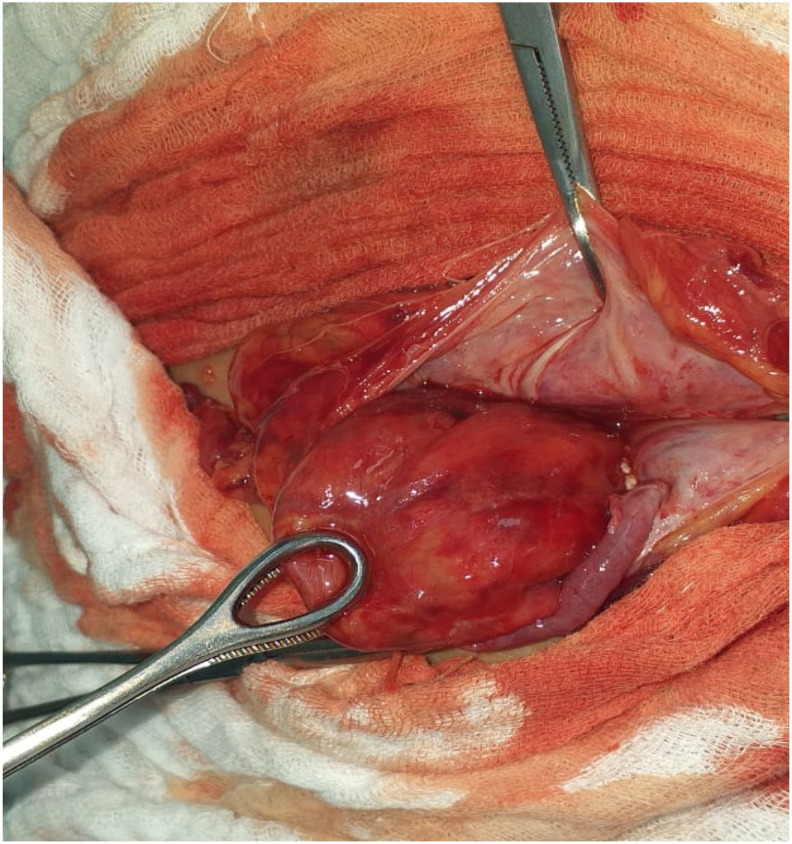
per operative showing the contain of the peritoneal sac.

Forty two year old man, with diabetic mellitus treated with oral antidiabetics over 8 years, hypertension over 5 years under treatment, operated for left hydrocele 3 years, consulted in emergency for a swelling of the right iliac fossa which appeared 5 years ago and became painful impulsive with and irreducible 5 days before consultation associated with vomiting and occlusive syndrome. The physical examination found an apyretic patient, distended abdomen with the presence of a painfull mass of 8 × 5cm in the right iliac fossa, irreducible. The pelvic digital examination revealed an empty rectal ampulla. Biology assessments found a hyperleukocytosis with 12550 white blood cells/ml. The diagnosis of a strangulated spiegel hernia was retained. Surgical exploration, by local incision, found an 8cm hernial sac with a 2cm hole at the junction of the lateral edge of the rectus maximus and the transverse muscles of the abdomen with the caecum and appendix herniation. An appendectomy was performed and the caecum moved back in the abdominal cavity after resuscitation. The peritoneal sac was resected. Closure of the orifice by a 2 resorbable interrupted suture was performed (Fig. 1)

Closure of the aponeurosis of the external oblique muscle with interrupted stitches with absorbable thread. A subcutaneous redon drain was installed and the postoperative was simple. The patient was discharged on the third postoperative day after transit retake and drain removal. the patient was seen after one month in a good health.

## 2 Discussion

Spiegel's hernias are rare and correspond to the protrusion of a peritoneal sac through an acquired or congenital anatomical orifice of the Spiegel's line located at the crossing of the fibres of the transverse and oblique abdominal muscles on the lateral edge of the rectus abdominis [3]. This weak point is limited at the bottom by the lower epigastric artery. Spiegel's hernias are initially located behind the rectus muscle and are externalized at its outer edge along an oblique path forwards and outwards. The peritoneal sac progressively slides below the arcuate line. Most hernias thus occur at the level of the Spiegelian girdle, an area 6 cm high situated between the umbilicus at the top and a line passing through the anterior superior iliac spines at the bottom [4]. There are predisposing factors such as intra-abdominal hyperpressure secondary to morbid obesity, multiple pregnancies and chronic cough [5]. The neck of the hernia is usually 0.5–2 cm narrow, and is therefore responsible for incarcerations and strangulations with occlusive syndrome [6] clinical diagnosis of Spiegel's hernias is difficult. In the constituted forms, all the aponeurotic planes are repressed and the diameter of the hole can be important it represents less than 1% in a series of 117 patients ([7] incidence of strangulation and incarceration of this type of hernia is estimated at 17%, requiring urgent surgical intervention [7]. The abdominal CT scan, has great sensitivity, remains the key examination of the diagnosis of the hernia and its contents [8]. Treatment is surgical; Different approaches are feasible: direct, by raphy or pre-peritoneal or pre-aponeurotic prosthetic replcement; laparoscopic, by intra-peritoneal, *trans*-abdominal-preperitoneal or extra-peritoneal raphy or mesch repair. If the hernial ring is narrow (less than 2 cm) A direct approach herniorrhaphy is usually sufficient. Otherwise, a laparotomy approach or transperitoneal laparoscopic repair with a double-sided or pre-peritoneal mesch are performed, with a lower risk of recurrence with synthetic mesch than simple suture [9].

## 3 Conclusion

Spiegel hernia is a rare pathology. Strangulation remains a rare but serious complication that can be life-threatening. Abdominal CT scan allows the diagnosis and reveals complications, in particular of strangulated hernia.

## Ethical approval

As per international standard written ethical approval has been collected and preserved by the author(s).

## Sources of funding

None.

## Authors' contributions

This work was carried out in collaboration among all authors. All authors contributed to the conduct of this work. They also declare that they have read and approved the final version of the manuscript.

## Guarantor

DR EL ATTAR LAYLA.

## Consent

Written informed consent was obtained from the patient for publication of this case report and accompanying images. A copy of the written consent is available for review by the Editor-in-Chief of this journal on request.

## Registration of research studies

Researchregistry2464.

## Declaration of competing interest

Authors have declared that no competing interests exist.

## References

[1] Mittal T., Kumar V., Khullar R. (2008). Diagnosis… - google scholar. https://scholar.google.fr/scholar?hl=fr&as_sdt=0%2C5&q=Mittal+T%2C+Kumar+V%2C+Khullar+R%2C+et+al+%282008%29+Diagnosis+and+management+of+Spigelian+hernia%3A+A+review+of+literature+and+our+experience.+J+Minim+Access+Surg+4%3A95%E2%80%938&btnG=.

[2] Moszkowicz D., Paye F., Balladur P., Lefevre J.H. (2012). Une cause rare d’occlusion aiguë du grêle : la hernie de Spiegel étranglée. Description d’un cas et revue de la littérature. Morphologie. 1 mars.

[3] Agha R.A., Franchi T., Sohrabi C., Mathew G., Kerwan A., Thoma A. (2020). The SCARE 2020 guideline: updating consensus surgical CAse REport (SCARE) guidelines. Int J Surg. déc.

[4] Skandalakis P.N., Zoras O., Skandalakis J.E., Mirilas P. (2006). Spigelian hernia: surgical anatomy, embryology, and technique of repair. Am. Surg..

[5] Liberge R., Frampas E., Madoz A., Leaute F., Dupas B. - google scholar. https://scholar.google.fr/scholar?hl=fr&as_sdt=0%2C5&q=Liberge+R%2C+Frampas+E%2C+Madoz+A%2C+Leaute+F%2C+Dupas+B.+Imagerie+des+hernies+pari%C3%A9tales+abdominales.+Datatraitesr433-61105+%5BInternet%5D.+2014+Sep+30&btnG=.

[6] Marschall J., Vergis A. (2004). Radiology for the surgeon: soft-tissue case 53. Can. J. Surg..

[7] Moles Morenilla L, Docobo Durantez F, Mena Robles. - Google scholar [Internet]. [cité 3 févr 2021]. Disponible sur https://www.sciencedirect.com/science/article/pii/S2049080121004039.

[8] Une hernie étranglée sur le fascia de Spiegel | SpringerLink. https://link.springer.com/article/10.1007/s13341-017-0769-6#citeas.

[9] Akpo G., Deme H., Badji N., Niang F., Toure M., Niang I. Diagnostic tomodensitométrique d’une hernie de Spiegel étranglée: à propos d’une observation, Pan Afr Med J. https://www.ncbi.nlm.nih.gov/pmc/articles/PMC5337274/.

